# Identifying Unmet Treatment Needs for Patients With Osteoporotic Fracture: Feasibility Study for an Electronic Clinical Surveillance System

**DOI:** 10.2196/jmir.9477

**Published:** 2018-04-24

**Authors:** Fong-Ci Lin, Chen-Yu Wang, Rung Ji Shang, Fei-Yuan Hsiao, Mei-Shu Lin, Kuan-Yu Hung, Jui Wang, Zhen-Fang Lin, Feipei Lai, Li-Jiuan Shen, Chih-Fen Huang

**Affiliations:** ^1^ Graduate Institute of Biomedical Electronics and Bioinformatics National Taiwan University Taipei Taiwan; ^2^ Department of Pharmacy National Taiwan University Hospital Taipei Taiwan; ^3^ Graduate Institute of Clinical Pharmacy College of Medicine National Taiwan University Taipei Taiwan; ^4^ School of Pharmacy College of Medicine National Taiwan University Taipei Taiwan; ^5^ Information Technology Office National Taiwan University Hospital Taipei Taiwan; ^6^ Department of Development and Planning National Taiwan University Hospital Taipei Taiwan; ^7^ Department of Internal Medicine National Taiwan University Hospital Taipei Taiwan; ^8^ Department of Internal Medicine National Taiwan University Hospital Hsinchu Branch Hsinchu Taiwan; ^9^ Institute of Epidemiology and Preventive Medicine College of Public Health National Taiwan University Taipei Taiwan; ^10^ Department of Computer Science & Information Engineering National Taiwan University Taipei Taiwan; ^11^ Department of Electrical Engineering National Taiwan University Taipei Taiwan

**Keywords:** information systems, public health surveillance, osteoporotic fractures, pharmacovigilance, guideline adherence

## Abstract

**Background:**

Traditional clinical surveillance relied on the results from clinical trials and observational studies of administrative databases. However, these studies not only required many valuable resources but also faced a very long time lag.

**Objective:**

This study aimed to illustrate a practical application of the National Taiwan University Hospital Clinical Surveillance System (NCSS) in the identification of patients with an osteoporotic fracture and to provide a high reusability infrastructure for longitudinal clinical data.

**Methods:**

The NCSS integrates electronic medical records in the National Taiwan University Hospital (NTUH) with a data warehouse and is equipped with a user-friendly interface. The NCSS was developed using professional insight from multidisciplinary experts, including clinical practitioners, epidemiologists, and biomedical engineers. The practical example identifying the unmet treatment needs for patients encountering major osteoporotic fractures described herein was mainly achieved by adopting the computerized workflow in the NCSS.

**Results:**

We developed the infrastructure of the NCSS, including an integrated data warehouse and an automatic surveillance workflow. By applying the NCSS, we efficiently identified 2193 patients who were newly diagnosed with a hip or vertebral fracture between 2010 and 2014 at NTUH. By adopting the filter function, we identified 1808 (1808/2193, 82.44%) patients who continued their follow-up at NTUH, and 464 (464/2193, 21.16%) patients who were prescribed anti-osteoporosis medications, within 3 and 12 months post the index date of their fracture, respectively.

**Conclusions:**

The NCSS systems can integrate the workflow of cohort identification to accelerate the survey process of clinically relevant problems and provide decision support in the daily practice of clinical physicians, thereby making the benefit of evidence-based medicine a reality.

## Introduction

Clinical surveillance can provide information on disease prognosis and postmarketing medication safety, which helps researchers identify potential clinical issues [1,2]. We describe the development and implementation of a Web-based National Taiwan University Hospital Clinical Surveillance System (NCSS) that is an interoperable, reusable, and scalable platform for collaborative data sharing using the ASP.Net framework (Microsoft, WA, USA).

Traditional clinical surveillance relied on the results from clinical trials and observational studies of administrative databases. However, these studies not only required a lot of valuable resources but also faced a very long time lag. Several studies [3-15] have described the difficulty of reducing gaps between clinical research needs and proper data management technique. Therefore, we aimed to develop an automated system with the capability to use routinely collected electronic health care data to support clinical surveillance is an important issue.

The National Taiwan University Hospital launched a new 3-year strategic plan to build the NCSS in 2015. Therefore, we reviewed the literature to gain insights into the challenges of cross-networking, patient privacy, user-friendliness, and reusability (Table 1). To achieve data harmonization, we referenced the common data model from the Sentinel [4,5]. In addition, the PCORnet [3,9] developed a common data model derived from the Sentinel [4,5] to support the development of an analyzable research database that enhanced the performance of a cross-networking query. We now need to consider the system architectural and data management structures. The Research Electronic Data Capture [14] developed reusable tools for project-specific clinical and translational research data. We found that the design of the Stanford Translational Research Integrated Database Environment [15] for an anonymous patient cohort discovery tool provides a flexible research data management solution. In addition, the Integrating Biology and the Bedside [13] proposed a self-scaling, interoperable platform for collaborative data sharing. Finally, the Observational Health Data Sciences and Informatics is an international collaboration on open-source data analytic solutions [12].

Nonetheless, only a few successful efforts for high reusability and computerized workflow infrastructure have been accomplished in Taiwan to date. Therefore, we decided to implement a Web-based clinical surveillance system extensible to interdisciplinary collaboration and data sharing.

Our efforts are based on 2 highly diffusible, highly reusable, and thin client architectures for research network. The platform provides a high reusability infrastructure for a computerized workflow that captures relevant longitudinal clinical data and makes those data repositories reusable. Finally, the platform has been used for multi-municipality surveillance.

**Table 1:** Literature summary on electronic clinical surveillance, sorted by year of publication.

Author	Year	Main concept	Country
Brown et al [4,5]	2010	Data harmonization	United States
Obeid et al [14]	2013	Reusable tools for project-specific clinical and translational research data	International
Natter et al [13]	2013	A self-scaling, interoperable platform for collaborative data sharing	United States
Lowe et al [15]	2014	An anonymous patient cohort discovery tool and data management solution	United States
Waitman et al [3] and Fleurence et al [9]	2014	An analyzable research database that enhanced performance of a cross-networking query	United States
Hripcsak et al [12]	2015	An international collaboration on open-source data analytic solutions	International

## Methods

### Data Warehouse

The NCSS integrates a database of electronic medical records at National Taiwan University Hospital (NTUH), which is a medical center in Taiwan with over 2000 beds. The clinical data models were built using an Oracle 11g relational database; included the demographics, diagnosis, pharmacies, procedures, laboratories, and death records; and were implemented into the data warehouse process.

The data warehouse process is the collection of electronic medical records from the Integrated Medical Database (IMDB) through scheduling using an extraction, transformation, and loading tool. We refreshed the database during nonbusiness hours using 3 steps. First, the system extracted data changes by comparing the time difference with IMDB. Second, personal identifiable information was fully anonymized. Finally, the data are synchronized back to the IMDB with a timestamp. This data warehouse provides a data access infrastructure for the NCSS.

### Workflow

We aimed to present a Web-based NCSS for clinical surveillance in a secure, efficient, and interoperable platform. The NCSS used ASP.Net framework (version 4.5, Microsoft) for Web development and cloud batch process. The NCSS is configured to run using load balancing, including failover modes, to secure the system’s availability and scalability. Firewalls were also installed to enhance the security of the NCSS. In addition, all queries were audited and logged, which assures compliance with institutional review board and other regulatory protections for subjects.

**Figure 1:**
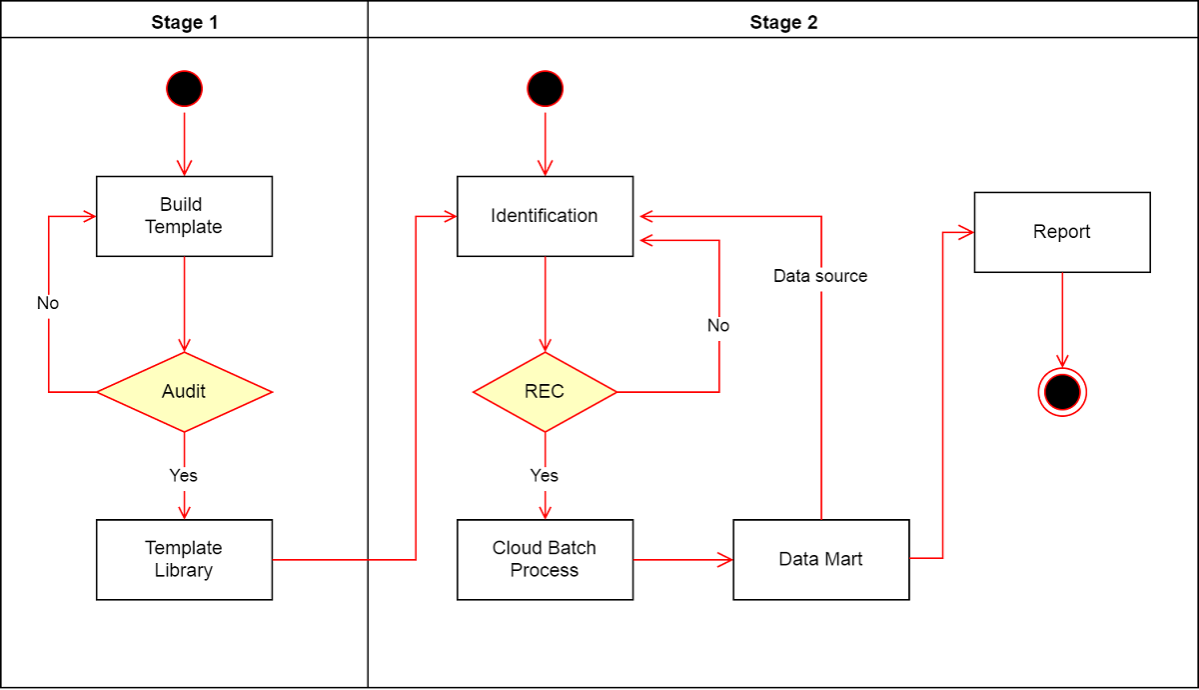
System workflow of the National Taiwan University Hospital Clinical Surveillance System (NCSS). REC: Research Institutional Ethics Committee.

We designed thin client architecture so that the researchers can focus on the research design. After setting the parameters on the webpage, the researcher can leave the large computing delegate to cloud. Therefore, the researchers do not need to own a good performance of the computer and lower the computation of big data of barriers. The overall workflow of the NCSS is depicted in Figure 1.

### Stage 1. Build a Template of Clinical Orders

The process supports the end user, typically a clinical researcher, to predefine a template of clinical orders using a Clinical Orders Navigator on the client side. The researcher can browse and search different dimensions, such as the diagnosis (International Classification of Diseases, ICD-9 or ICD-10), pharmacy (anatomical therapeutic chemical code), procedure, and laboratory in the integrated interface. The details of Clinical Orders Navigator are provided in Multimedia Appendix 1.

The stage can help clinical researchers build a protocol-based standardized process and save those clinical orders and specific guidelines to the database. The creator of the templates can choose to commit them to Template Library publicly. All templates submitted to the public Template Library will be reviewed by clinical professionals to ensure the quality and accuracy. All researchers can create their own template or use the open template applied to the Identification process.

### Stage 2. Patient Identification

The stage of patient identification includes matching of the clinical needs to the optimal cohort study. The stage contains 5 processes, consisting of identification, REC (Research Institutional Ethics Committee) verify, cloud batch process, data mart of patient level, and report service.

1. Identification: We developed an electronic form to meet the cohort study flow that contains 2 setting as input as follows (when researchers finished the electronic form, the system will save that setting to the database and generate a universally unique identifier as case number of this setting.):
  - Basic setting: In this step, the researcher fills the surveillance topic, and the duration of observation from a particular data source, such as outpatient, admission, and emergency. In addition, this setting also supports the selection of specific patient list from data mart (patient level).
  - Order setting: In this step, the researcher chooses the order setting in Clinical Orders Navigator. The clinical orders can be in different dimensions, such as the diagnosis, pharmacy, procedure, and laboratory. For example, a disease can be defined using several diagnosis codes, such as hip or vertebral fractures, including ICD-9-codes 820, 805, and 806 in the Template Library. The researchers can reuse these templates as include or exclude criteria to design their study flow.
2. REC verify: All settings of Identification need to be been verified by REC. If the setting had been authorized, it will add a task to the Task Queue for cloud batch process in server side.
3. Cloud batch process: The cloud batch process maintains a Task Queue. Every task will be executed follow First-In-First-Out mechanism in batch server that read the eligible patients in IMDB. The batch process also records the cost of each query and the number of patients identified. After completion of the task, the patient list will be stored into the database and a mail notification is will be sent to the researchers.
4. Data mart (patient level): The data mart is a collection of the patient list that presents the result of every identification process. The researcher can adopt a patient list from data mart as their patient data source to query the next Identification process. Therefore, the identification process can support a hierarchical structure. This means that the process can generate a new study population based on a previous screening result. The researchers can reuse these patient lists to design a cohort study or case control study in fine-grained categorization.
5. Report service: The report service contains 3 views of summary statistics for patient list in data mart as follows:
  - View of characteristics: The view is a demographic summary that helps clarify the characteristics for the patient list, for example, the descriptive statistics of age, gender, body mass index (BMI), and income level.
  - View of longitudinal incidence trend: The view presents incidence trend by time series chart and provides a real-time interactive query by time interval, including monthly, quarterly, or yearly.
  - View of source record: The view presents the number of included/excluded patients in every Identification process. If the patient list contains a hierarchical structure that runs the process of identification more than once, the report service can track all results of identification in the aggregation table.

### A Practical Example of a Clinical Application of the National Taiwan University Hospital Clinical Surveillance System in the Identification of Osteoporotic Fracture Patients

For a practical example of a clinical application of the NCSS, we looked at the identification of osteoporotic fracture patients and their utilization in pharmacological therapy. Osteoporotic fractures, a major consequence of osteoporosis, are associated with a high mortality rate, increased risk of re-fracture, and poor quality of life, and incur heavy economic burden on the society.

According to a report from the Global Burden of Disease Study project, the global burden of osteoporosis-related problems has doubled in the past two decades and has shown a continuous increase in recent years. In 2016, 441,230 documented deaths could be attributed to osteoporosis-related problems [16,17].

In the United States, the direct economic burden of osteoporotic fractures was approximately US $17 billion in 2005, and is projected to increase 50% by 2025 [18]. Fortunately, the safety and efficacy of anti-osteoporosis medications (AOMs) used by patients with established osteoporotic fractures have been ascertained [19-23]. However, despite the readily available and effective treatment for osteoporosis, a care gap between established osteoporotic fractures and the pharmacological prevention of subsequent fractures is still being discussed worldwide [24-27]. We aimed to identify the unmet treatment needs for patients encountering major osteoporotic fractures with the NTUH-based clinical surveillance system.

Using NCSS as our data source, we identified patients newly diagnosed with a hip (ICD-9-code 820) or vertebral fracture (ICD-9-codes 805 and 806) between 2010 and 2014 as our study subjects, and defined them as patients requiring treatment. The initial diagnosis date of a hip or vertebral fracture was defined as the index date of the study subject. Patients under 50 years of age; with a diagnosis of malignant neoplasm (ICD-9-code 140-208), osteoporotic fracture (ICD-9-codes 820, 805, and 806), or Paget disease (ICD-9-code 731.0); or who had been prescribed AOMs within 1 year before the index date were excluded. Among them, we investigated the prescription pattern of the AOMs by distinguishing patients into those who began taking an AOM within 1 year after the index date, and those who did not. The AOMs evaluated in this study included alendronate, zolendronate, ibandronate, denosumab, raloxifene, teriparatide, estradiol valerate, conjugated estrogens, and calcitonin. The system setting of the identification process used by the NCSS, the demographics of the study population, and the treatment pattern of the study population were presented quarterly.

## Results

**Figure 2:**
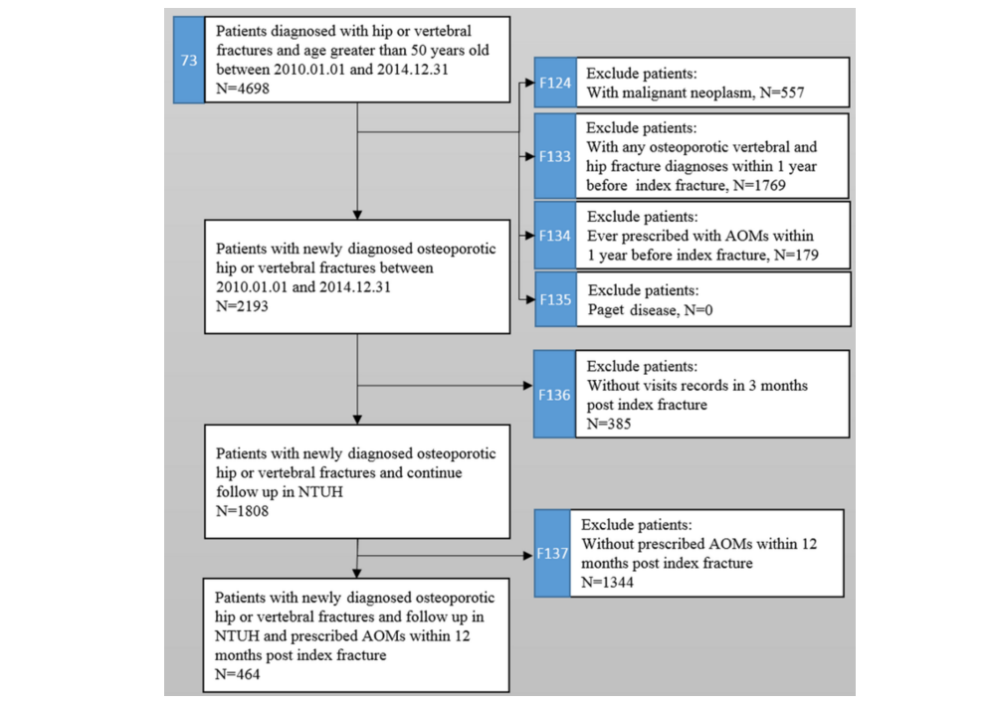
Study flowchart implemented by National Taiwan University Hospital Clinical Surveillance System (NCSS). Each identification process had been assigned a universally unique identifier with a case number (marked by the blue background, such as 73, F124, F133, F134, F135, F136, and F137). The case number with an F as a prefix stands for its’ own hierarchical structure. NTUH: National Taiwan University Hospital; AOMs: anti-osteoporosis medications.

The NCSS design uses a protocol-based standardized process of incremental development, testing, and deployment to meet specific clinical needs. We demonstrated a practical example of identifying the unmet treatment needs for patients encountering major osteoporotic fractures and implemented the hierarchical study population using the NCSS, as depicted in Figure 2.

**Figure 3:**
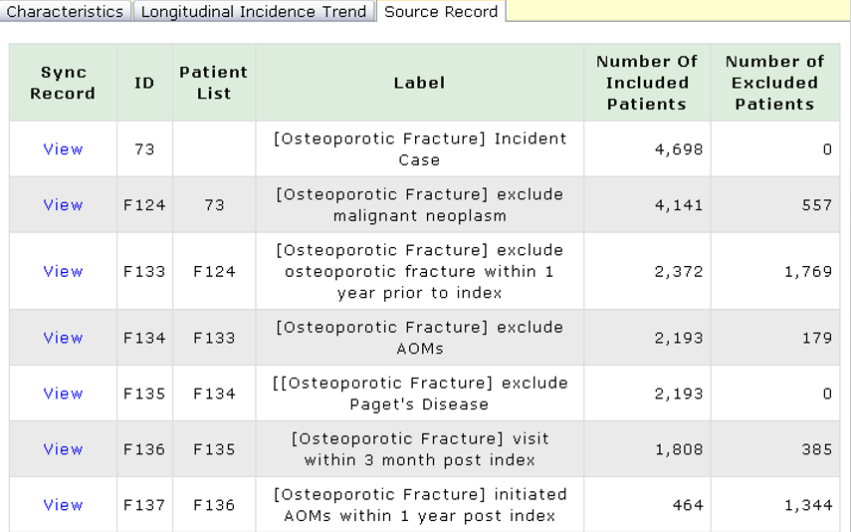
Snapshot of a source record in National Taiwan University Hospital Clinical Surveillance System (NCSS). Through the source record, we can get the number of included (and excluded) patients and the data source (patient list).

We initially selected older patients diagnosed with a hip or vertebral fracture between 2010 and 2014. By adopting the identification and filter function of the NCSS, patients with a history of malignant neoplasm (n=557), or osteoporotic vertebral and hip fracture (n=1769) within 1 year before the index date, were excluded. In addition, to identify a new AOM user, we excluded 179 patients with an AOM prescription before the index date. We identified 2193 incidence cases for hip or vertebral fractures within the period of 2010-2014. These patients were defined as “patients requiring treatment” according to the current treatment guidelines. In addition, in the Report Service, we used visualization tools for displaying the summary statistics for each patient list. This is depicted in Figure 3.

To ensure those participants are having a continuous follow-up, we excluded 385 patients who had not visited the hospital within 3 months after the index date. We enrolled in the study 1808 patients who had continued to follow-up at NTUH within 3 months post the index date of their fracture. To investigate the prescription pattern of the AOMs, we established 2 groups that were classified based on their AOM prescription date, and by adopting a filter function, we identified 1808 (1808/2193, 82.44%) patients who continued to follow-up at NTUH, and 464 (464/2193, 21.16%) patients prescribed with an AOM, within 3 and 12 months post the index date of their fracture, respectively.

**Figure 4:**
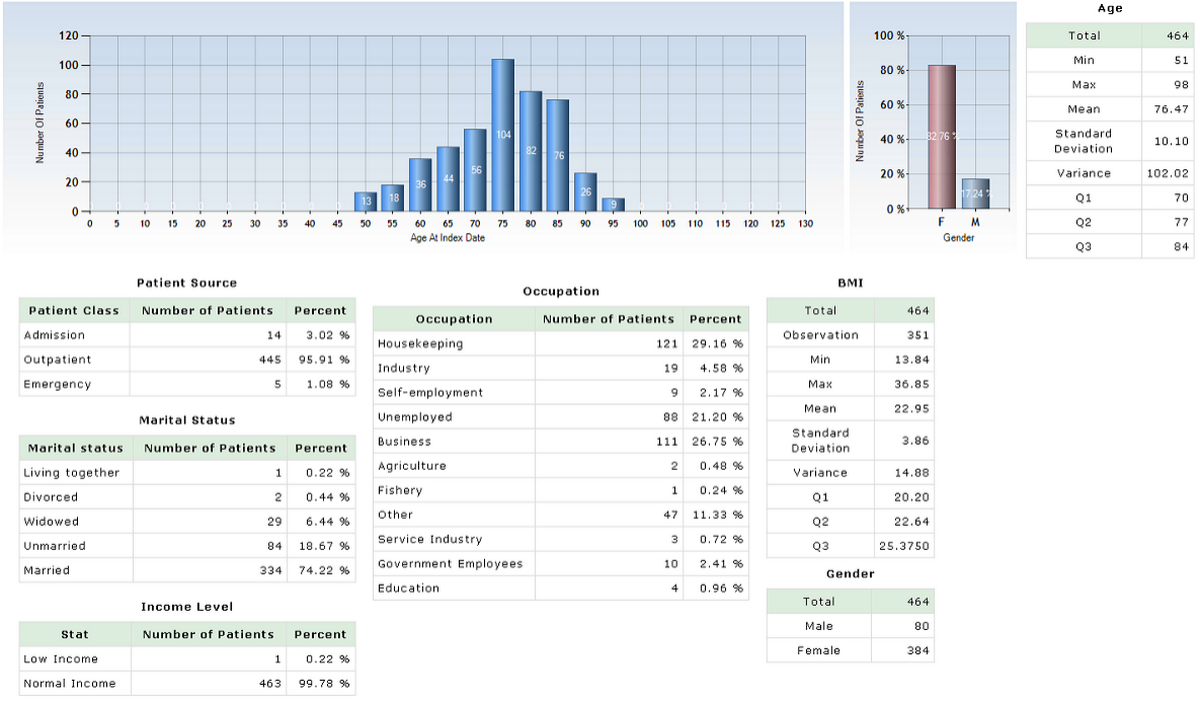
Snapshot of Report of characteristics in the National Taiwan University Hospital Clinical Surveillance System (NCSS).

The NCSS provided a summary of the baseline characteristics of the study population including gender, age, BMI, socioeconomic status, occupation types, and marital status, as shown in Figure 4. For example, among the patients who began taking an AOM within 1 year after the index date, their mean age was 76.47 (SD 10.10) and their mean BMI was 22.95 (SD 3.86) kg/m^2^; in addition, the proportion of females was 82.7% (384/464). This population showed a high proportion of married patients 71.98% (334/464) with a normal income level 99.8% (463/464). NCSS provided information regarding the drug utilization of AOMs for the study population.

**Figure 5:**
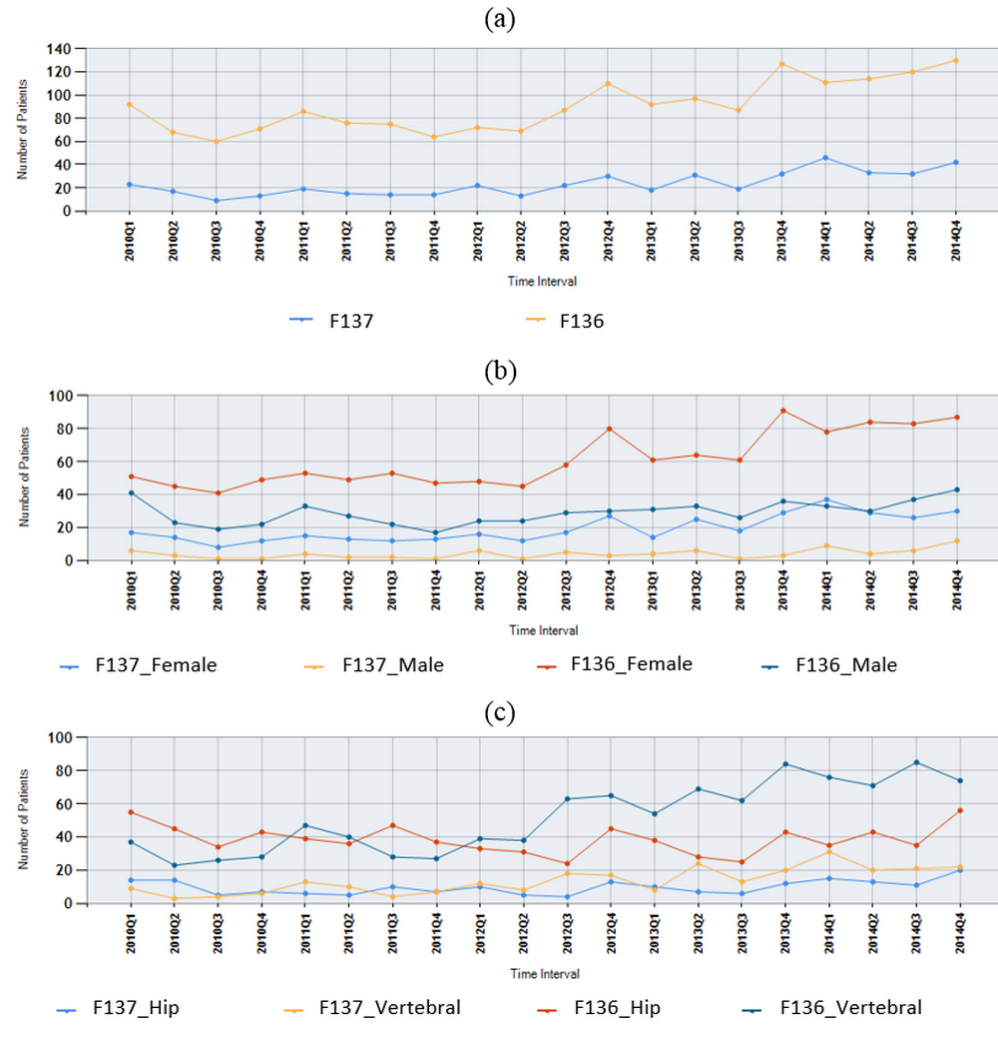
Snapshot of the quarterly report for incidence trend of drug utilization of study cohorts.

The longitudinal trends of patients newly diagnosed with an osteoporotic fracture and those who began taking an AOM within 1 year after the index date of their fracture are illustrated in Figure 5. For example, taking the information from Figure 5, we found that there were approximately 130 newly diagnosed osteoporotic fracture patients continuing their follow-up at NTUH in Quarter 4 2014, and among them, 42 (42/130, 32.3%) began taking an AOM within 1 year post the index date of their fracture.

Furthermore, the NCSS provides the choice of information stratification based on gender, index date, fracture type, and other types of information, which can be provided monthly, quarterly, and yearly, thereby increasing the flexibility of the clinical interpretation. For example, as shown in part (b) of Figure 5, there were 87 female and 43 male fracture patients in 2014 Q4, and among them, 30 (30/87, 35%) females and 12 (28%, 12/43) males began taking an AOM within 1 year post the index date of their fracture. Information on patients with different fracture types can be seen in part (c) of Figure 5.

## Discussion

### Principal Findings

To the best of our knowledge, NCSS is a pioneering electronic clinical surveillance system in its attempt to organize decision-making activities and facilitate the standard-based process for clinical needs in Taiwan. In this study, we demonstrated a practical example of an NCSS application. We can efficiently and correctly gather information on those patients requiring disease treatment and understand their treatment patterns, as well as identify any unmet treatment requirements in the hospital.

Through this practical example, we found that the pharmacological treatment rate of patients with an osteoporotic fracture is suboptimal at NTUH. On average, only 35% (30/87) of female and 28% (12/43) of male osteoporotic fracture patients initiated AOM therapy to prevent a subsequent fracture. More effort is warranted to improve the quality of care for these patients.

### Limitations

Studies from a hospital-wide database require less time for data collection and can achieve a faster response to meet the needs of clinical research. However, there is a certain limitation to this study, that is, bias in the estimates may exist because it is difficult to determine whether the patient in a hospital-wide database is suffering from such injury for the first time.

### Comparison With Prior Work

Clinical surveillance systems have been widely implemented [13,15,28], and studies have demonstrated the use of various algorithms to identify potential research patient cohorts. There are several important core concepts to our research, including access to clinical data for research purposes, the design of a flexible research data management solution, and clarifying the characteristics of each study population. However, previous studies [8,9,13,15] have not proposed how to integrate a protocol-based process in the building of a cohort discovery. The clinical surveillance systems should also have the capability to bring guidelines to the clinical practitioners. The NCSS integrates the workflow of cohort identification to accelerate the survey process based on certain guidelines. In particular, for quality assurance, clinical researchers or medical policy makers need to monitor specific quality indicators to ensure the quality of patient care. They can focus on clinical needs to achieve a continuous process integration using standardized NCSS templates. The different clinical contexts can be refined using a new scaffold to meet clinical needs based on the original standardized templates. In fact, different methodologies of capturing cases of patients would result in disparate estimates of incidence or mortality. For example, in the case on sepsis identification, there are four methods available, [29-32] including Angus et al, Martin et al, Dombrovskiy et al, and Wang et al. We believe this clinical knowledge should be preserved and converted into a shareable template for a collaborative research network. These templates can be quickly searched and reused for inclusion/exclusion criteria of patient identification in the Template Library. Therefore, the researchers can embrace change courageously because they only need to focus on any existing differences. More importantly, these processes should be conceptualized as continuous organizational efforts to lead to self-organizing innovation of the NCSS.

The hierarchical structure of the system design has not been proposed for system reusability. We designed a mechanism for reusing the patient list in data mart. We believe this identified list of patients can be reused by other researchers, especially for the design of a subgroup study, a case control study, or a similar context of research. To better interpret the process of identification of each patient list, we designed a hierarchical structure where each patient list could be traced back to its patient data source and researchers could compare every patient data source with characteristics and longitudinal incidence trend in the Report Service. This innovative method allows dynamically generating a clinical data mart and reduces the computation overhead through the reuse of the same patient list. This design can inspire researchers and allow them to focus on the research design rather than data processing.

Until 2017, the NCSS has been well constructed and continuously improving. The active users of NCSS are now in the form of research teams. These teams consist of multidisciplinary specialties such as a clinical doctor, pharmacist, epidemiologist, bio-medical engineer, and research assistants. Several research teams have been adopting the NCSS to improve the problem-solving process of their relevant clinical issue. This includes cardiovascular, diabetic, renal, liver, neurologic, and orthopedic medical teams. Each team consists of 5-7 people. These research teams regularly hold monthly meetings to discuss the problems related to the application of NCSS. In the period between 2015 and 2017, dozens of cohorts were created by each research team. Moreover, a large sum of meaningful feedback has been received for not only the problem related to using the NCSS but also recommendations for improving the NCSS. For example, the cardiovascular research team raised the problem of using different brands of drugs during different periods. Therefore, we retrieved the relevant drug products available and used in 2014 as well as 2004. This modification allows the research to more precisely capture and study the drug exposure across a population. The orthopedic research team also revealed the problems regarding the inconsistent days listed for a prescription and the pharmacological duration of the prescription. Some medication has a 1 year of effect duration; however, the prescription was recorded for only 1 day. Therefore, the NCSS modified the duration according to each drug property to correct the measurement of exposure. Finally, we accomplished the assistant resources such as the user handbook and Web-based video tutorials. This helps the research team readily assess the NCSS. These application experiences and associated feedback help to improve the NCSS efficiency and quality of clinical research at NTUH.

### Future Work

The identification of the problem is the first step to solving and improving the clinical outcome of the patient. By applying computerized patient identification derived from the NCSS, we can create the infrastructure of an informatics system at NTUH. Furthermore, we can provide decision support in daily practice, thereby making the benefit of evidence-based medicine a reality. In fact, we believe that the best way to promote medical care is to provide relevant evidence to assist doctors in their decision-making process during their clinical daily practice. For example, an evaluation of the longitudinal trends of health care utilization can help create a baseline, track progress over time, and generate real-world evidence. Besides providing clinical support for physicians, the next step will be providing integrated real-time, interactive, and personalized support to individual patients [33,34]. This will be focused on in a future study. Finally, we strongly advocate developing a consistent strategy, as well as celebrating success and continuously sharing different experiences. The continual reduction in the gap between evidence and practice is an ongoing journey and not an end that can be simply reached shortly after the NCSS is implemented.

### Conclusions

The results of this study confirm that the NCSS is an efficient electronic clinical surveillance system that can integrate the workflow of cohort identification to accelerate the survey process of disease and medication prescription patterns. The NCSS can serve the critical role of gathering data and making associations between evidence and practice in a rapid fashion, and can be a support system for researchers who wish to confirm certain clinical issues as well as for those requiring a computerized system to complete their studies. Finally, the NCSS can provide decision support in the daily practice of clinical physicians and make the benefit of evidence-based medicine a reality.

## Appendix

Multimedia Appendix 1 A real-world use‐case deployed at National Taiwan University Hospital Clinical Surveillance System.

## Abbreviations

AOMs: anti-osteoporosis medications
BMI: body mass index
ICD: International Classification of Diseases
IMDB: Integrated Medical Database
NCSS: National Taiwan University Hospital Clinical Surveillance System
NTUH: National Taiwan University Hospital
REC: Research Institutional Ethics Committee
