# Supplementary material for: Identifying Unmet Treatment Needs for Patients With Osteoporotic Fracture: Feasibility Study for an Electronic Clinical Surveillance System
Source: J Med Internet Res. 2018 Apr 24;20(4):e142. doi: 10.2196/jmir.9477 (PMC5941097; doi:10.2196/jmir.9477)
Supplement: Multimedia Appendix 1 [file jmir_v20i4e142_app1.pdf]

APPENDIX:

Purpose: The intention of this appendix is to show a real-world use-case deployed at NCSS. The document contain 4 section, as following:

1. MODULE I: Clinical Orders Navigator

   Use this module to browse and search different dimensions in the integrated interface, such as the diagnosis (ICD-9 or ICD-10), pharmacy (ATC code), procedure and laboratory. In addition, the module supports end user to find public templates which was defined a set of clinical orders contributed by other researchers. We demonstrate five practical example, including how to find the clinical orders through full-text search and fuzzy search.

2. MODULE II: Orders Components

   This module can synchronize clinical orders from MODULE I: Clinical Orders Navigator. Many clinical orders are available for defining cohorts. This module support the end users to know the detail of clinical orders which they selected.

3. Case I: [Osteoporotic Fracture] Incident Case

   In the Case I, we will demonstrate a practical examples of system workflow in the NCSS. First, we introduce how to build a template and adopt the template for

patient identification. Second, we reveal how to use the Report Service that help clarify the characteristics for patient list.

4. Case II: [Osteoporotic Fracture] exclude malignant neoplasm

We will continue the results of case 1 and to carry out the process of patient identification in Case II. In this way, you can better understand the advantages of hierarchical structure.

Contents

MODULE I: Clinical Orders Navigator....2

Example 1: To retrieval the diagnosis codes for type I and type II diabetes mellitus....5

Example 2: To retrieval the diagnosis codes for type I diabetes mellitus....6

Example 3: To retrieval the drugs that affecting bone structure and mineralization by ATC-Code....7

Example 4: To retrieval the drug that indications is osteoporosis....8

Example 5: To find the template of Charlson Comorbidity Index....9

MODULE II: Orders Components....10

Case I: [Osteoporotic Fracture] Incident Case....12

Case II: [Osteoporotic Fracture] excluding malignant neoplasm....20

MODULE I: Clinical Orders Navigator

GOAL: Design a clinical orders search interface to supports researchers define which orders they want, intuitively. The navigator integrates multiple coding system, such as diagnosis (ICD-9), pharmacy (ATC Code), and laboratory (LOINC).

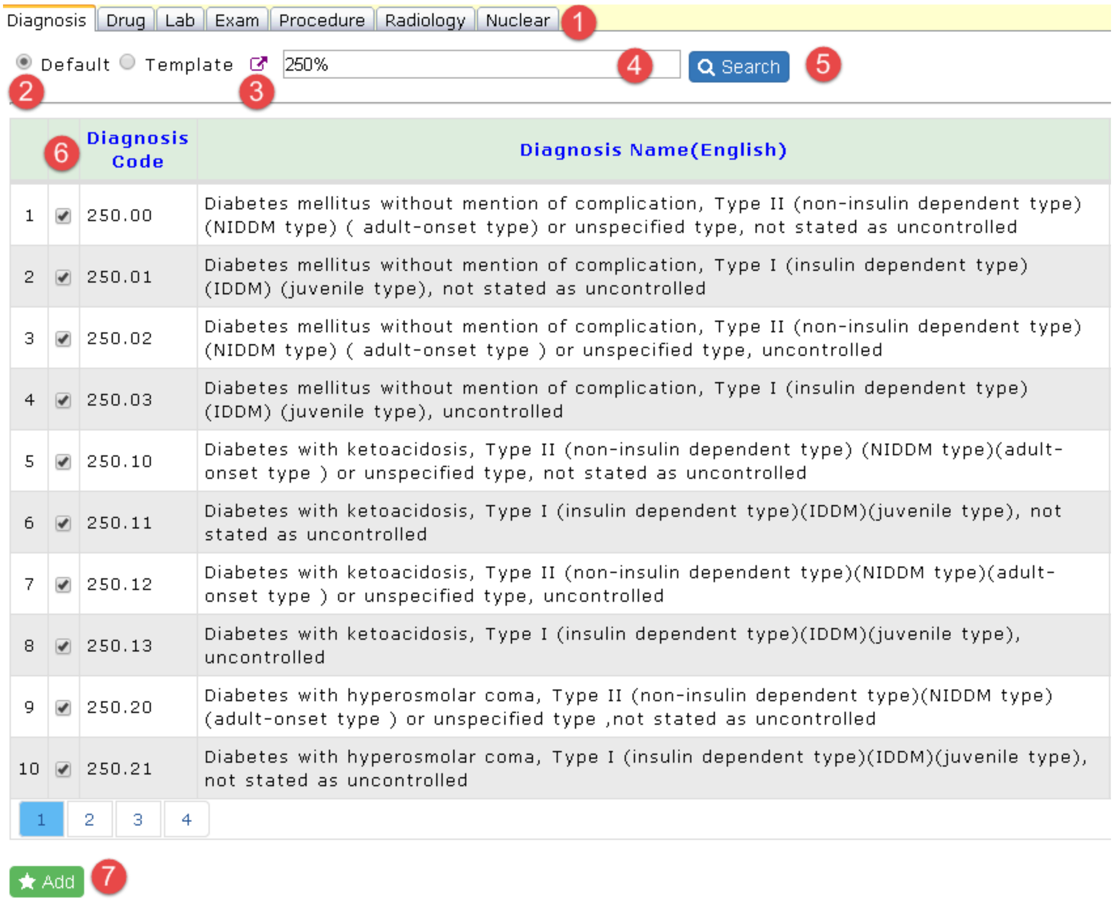

		Diagnosis Code	Diagnosis Name(English)
1	✔	250.00	Diabetes mellitus without mention of complication, Type II (non-insulin dependent type) (NIDDM type) (adult-onset type) or unspecified type, not stated as uncontrolled
2	✔	250.01	Diabetes mellitus without mention of complication, Type I (insulin dependent type) (IDDM) (juvenile type), not stated as uncontrolled
3	✔	250.02	Diabetes mellitus without mention of complication, Type II (non-insulin dependent type) (NIDDM type) (adult-onset type) or unspecified type, uncontrolled
4	✔	250.03	Diabetes mellitus without mention of complication, Type I (insulin dependent type) (IDDM) (juvenile type), uncontrolled
5	✔	250.10	Diabetes with ketoacidosis, Type II (non-insulin dependent type) (NIDDM type)(adult-onset type) or unspecified type, not stated as uncontrolled
6	✔	250.11	Diabetes with ketoacidosis, Type I (insulin dependent type)(IDDM)(juvenile type), not stated as uncontrolled
7	✔	250.12	Diabetes with ketoacidosis, Type II (non-insulin dependent type)(NIDDM type)(adult-onset type) or unspecified type, uncontrolled
8	✔	250.13	Diabetes with ketoacidosis, Type I (insulin dependent type)(IDDM)(juvenile type), uncontrolled
9	✔	250.20	Diabetes with hyperosmolar coma, Type II (non-insulin dependent type)(NIDDM type) (adult-onset type) or unspecified type ,not stated as uncontrolled
10	✔	250.21	Diabetes with hyperosmolar coma, Type I (insulin dependent type)(IDDM)(juvenile type), not stated as uncontrolled

Figure 1. Image of Clinical Orders Navigator

1. Clinical Order Type:

The researcher can choose different clinical order types include diagnosis, drug, laboratory and so on.

2. Search Mode:

Two mode can be chose: **Default Mode** and **Template Mode**. If the researcher wants to define a set of clinical orders by himself, he can choose the default mode. In addition, researcher can also use the public templates by choosing template mode which was defined a set of clinical orders contributed by other researchers.

3. Jump to Template Library:

If you want to browse which public templates are available, you can click the hyperlink to navigate the **Template Library**.

4. Query Keywords:

In order to improve the accuracy and flexibility for clinical orders search, in NCSS, support full text search and fuzzy search with wildcards.

%: Allows you to match any string of any length

_: Allows you to match any string of one length

5. **Search Button:**

To run the search based on the input values of **Query Keywords** and **Clinical Order Type**.

6. **Query Result:**

Researchers can click the checkbox in the table which presents the search result to decide which clinical orders they selected.

7. **Add Button:**

Synchronize clinical orders to **MODULE II Orders Components** according to the clinical orders they selected.

Example 1: To retrieval the diagnosis codes for type I and type II diabetes mellitus

- **Case:** fuzzy search with ‘%’
- **Order Type**: Diagnosis
- **Mode:** Default
- **Key Words**: 250%
- **Result**: query result show 40 records

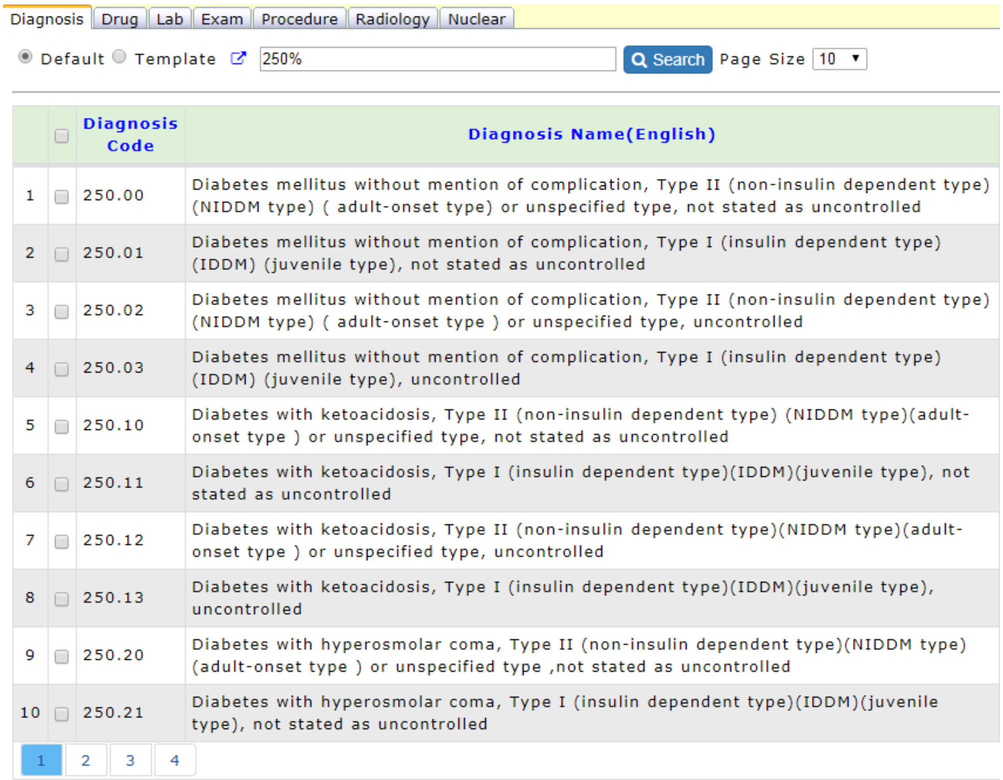

		Diagnosis Code	Diagnosis Name(English)
1		250.00	Diabetes mellitus without mention of complication, Type II (non-insulin dependent type) (NIDDM type) (adult-onset type) or unspecified type, not stated as uncontrolled
2		250.01	Diabetes mellitus without mention of complication, Type I (insulin dependent type) (IDDM) (juvenile type), not stated as uncontrolled
3		250.02	Diabetes mellitus without mention of complication, Type II (non-insulin dependent type) (NIDDM type) (adult-onset type) or unspecified type, uncontrolled
4		250.03	Diabetes mellitus without mention of complication, Type I (insulin dependent type) (IDDM) (juvenile type), uncontrolled
5		250.10	Diabetes with ketoacidosis, Type II (non-insulin dependent type) (NIDDM type)(adult-onset type) or unspecified type, not stated as uncontrolled
6		250.11	Diabetes with ketoacidosis, Type I (insulin dependent type)(IDDM)(juvenile type), not stated as uncontrolled
7		250.12	Diabetes with ketoacidosis, Type II (non-insulin dependent type)(NIDDM type)(adult-onset type) or unspecified type, uncontrolled
8		250.13	Diabetes with ketoacidosis, Type I (insulin dependent type)(IDDM)(juvenile type), uncontrolled
9		250.20	Diabetes with hyperosmolar coma, Type II (non-insulin dependent type)(NIDDM type) (adult-onset type) or unspecified type ,not stated as uncontrolled
10		250.21	Diabetes with hyperosmolar coma, Type I (insulin dependent type)(IDDM)(juvenile type), not stated as uncontrolled

1 2 3 4

Figure 2. Image of search result for Example 1 in Clinical Orders Navigator

Example 2: To retrieval the diagnosis codes for type I diabetes mellitus

- **Case**: fuzzy search with ‘_’
- **Order Type**: Diagnosis
- **Mode:** Default
- **Key Words**: 250._1
- **Result**: query result show 10 records

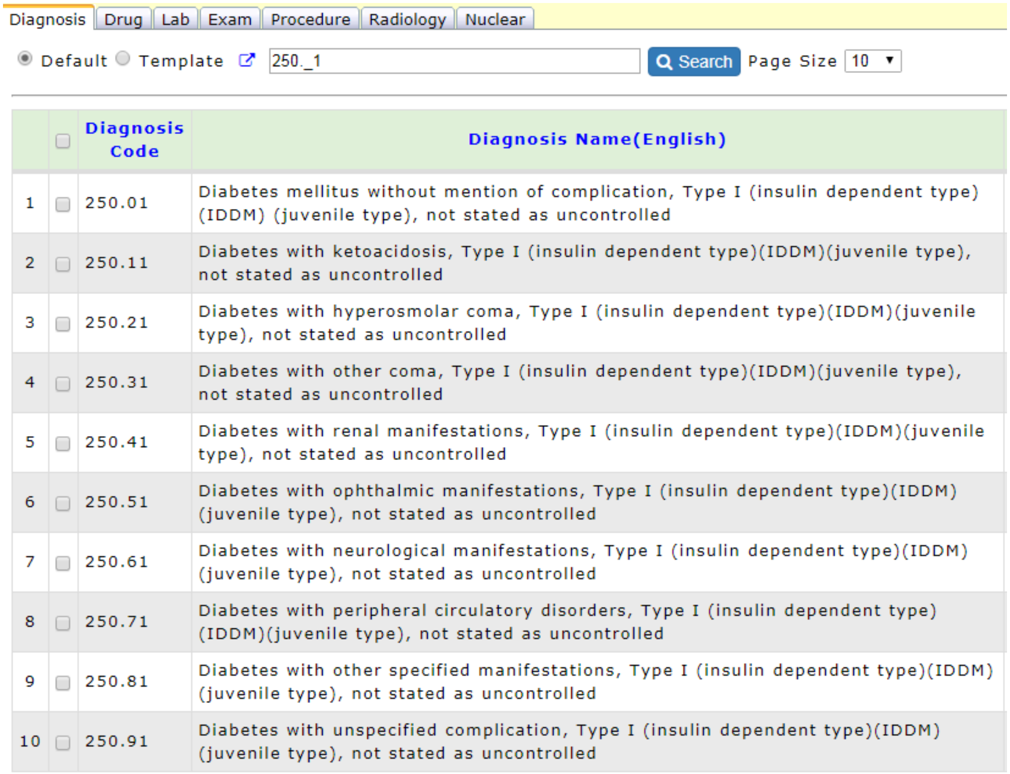

		Diagnosis Code	Diagnosis Name(English)
1		250.01	Diabetes mellitus without mention of complication, Type I (insulin dependent type) (IDDM) (juvenile type), not stated as uncontrolled
2		250.11	Diabetes with ketoacidosis, Type I (insulin dependent type)(IDDM)(juvenile type), not stated as uncontrolled
3		250.21	Diabetes with hyperosmolar coma, Type I (insulin dependent type)(IDDM)(juvenile type), not stated as uncontrolled
4		250.31	Diabetes with other coma, Type I (insulin dependent type)(IDDM)(juvenile type), not stated as uncontrolled
5		250.41	Diabetes with renal manifestations, Type I (insulin dependent type)(IDDM)(juvenile type), not stated as uncontrolled
6		250.51	Diabetes with ophthalmic manifestations, Type I (insulin dependent type)(IDDM) (juvenile type), not stated as uncontrolled
7		250.61	Diabetes with neurological manifestations, Type I (insulin dependent type)(IDDM) (juvenile type), not stated as uncontrolled
8		250.71	Diabetes with peripheral circulatory disorders, Type I (insulin dependent type) (IDDM)(juvenile type), not stated as uncontrolled
9		250.81	Diabetes with other specified manifestations, Type I (insulin dependent type)(IDDM) (juvenile type), not stated as uncontrolled
10		250.91	Diabetes with unspecified complication, Type I (insulin dependent type)(IDDM) (juvenile type), not stated as uncontrolled

Figure 3. Image of search result for Example 2 in Clinical Orders Navigator

Example 3: To retrieval the drugs that affecting bone structure and mineralization by ATC-Code.

- **Case**: fuzzy search with ‘%’
- **Order Type**: Drug
- **Mode**: Default
- **Key Words**: M058B%
- **Result**: query result show 14 records

Diagnosis | Drug | Lab | Exam | Procedure | Radiology | Nuclear

Default Template M05B% Search Page Size 20

		Drug Code	ATC Code	Generic Name	Brand Name (English)	Indications(English)
1		ACL1FAE2	M05BA08	Zoledronic acid	Aclasta 5mg	an inhibitor of osteoclastic bone resorption
2		ARE1FA06	M05BA03	Pamidronate Disodium	Aredia 15mg	Treats bone metastases of malignant tumors, malignant hypercalcemia and Paget's disease
3		BON1FA63	M05BA02	Sodium Clodronate	(DC)Bonefos 300mg	
4		BON1FAD8	M05BA06	Ibandronate Sodium	Bonviva 3mg	Treatment of osteoporosis in postmenopausal women
5		BON4FA63	M05BA02	Sodium Clodronate	Bonefos 400mg	Treat bone metastases of malignant tumors and malignant hypercalcemia
6		FOS4FAA5	M05BA04	Alendronate Sodium, Cholecalciferol	(DC)Fosamax Plus	Postmenopausal osteoporosis; Osteoporosis, in men
7		FOS4FAB1	M05BA04	Alendronate Sodium	(DC)Fosamax 70mg	
8		FOS4FAH7	M05BB03	Alendronate Sodium 70mg, Cholecalciferol 5600IU	Fosamax Plus	Postmenopausal osteoporosis; Osteoporosis, in men
9		FOS4FAH7	M05BA04	Alendronate Sodium 70mg, Cholecalciferol 5600IU	Fosamax Plus	Postmenopausal osteoporosis; Osteoporosis, in men
10		PAM1FA06	M05BA03	Pamidronate Disodium	Pamisol 15mg	Treats bone metastases of malignant tumors, malignant hypercalcemia and Paget's disease
11		PRO1FAG0	M05BX04	Denosumab	Prolia 60mg	Treats osteoporosis in men and postmenopausal women at high risk for fracture
12		XGE1FAG7	M05BX04	Denosumab	XGEVA 120mg	Prevention of skeletal-related events in patients with bone metastases from solid tumors.
13		ZOM1FAB5	M05BA08	Zoledronic Acid	Zometa 4mg	Treats high blood calcium levels and bone damage in patients with cancer.
14		ZOM1FAG8	M05BA08	Zoledronic Acid	Zometa 4mg	

Figure 4. Image of search result for Example 3 in Clinical Orders Navigator

Example 4: To retrieval the drug that indications is osteoporosis

- **Case**: full text search (full-text queries on the text in every column)
- **Order Type**: Drug
- **Mode**: Default
- **Key Words**: Osteoporosis
- **Result**: query result show 16 records

Diagnosis | Drug | Lab | Exam | Procedure | Radiology | Nuclear

Default Template Osteoporosis Search Page Size 20

		Drug Code	ATC Code	Generic Name	Brand Name (English)	Indications(English)
1		BON1FAD8	M05BA06	Ibandronate Sodium	Bonviva 3mg	Treatment of osteoporosis in postmenopausal women
2		CAL1FA35	H05BA01	Calcitonin, Salmon	Calcinin 50IU	Treats bone pain and other symptoms of Paget's disease. Treats osteoporosis and hypercalcemia.
3		EST4LF27	G03CA03	Estradiol Valerate	ESTRADE 2mg	Treats symptoms of menopause and lack of estrogen. Prevents osteoporosis after menopause.
4		EST4LF39	G03CA57	Conjugated Estrogens	Estromon 0.6250mg	Treats symptoms of menopause and lack of estrogen. Prevents osteoporosis after menopause.
5		EVI4LH04	G03XC01	Raloxifene HCl	Evista 60mg	Treats and prevents of osteoporosis in postmenopausal women .
6		FOR1FAD2	H05AA02	Teriparatide	(DC)Forteo 750mcg	Osteoprosis, Men with primary or hypogonadal osteoprosis or postmenopausal women with osteoporosis who are at high r
7		FOR1FAF3	H05AA02	Teriparatide	Forteo 600mcg	Osteoprosis, Men with primary or hypogonadal osteoprosis or postmenopausal women with osteoporosis who are at high r
8		FOS4FAA5	M05BA04	Alendronate Sodium, Cholecalciferol	(DC)Fosamax Plus	Postmenopausal osteoporosis; Osteoporosis, in men
9		FOS4FAA5	A11CC05	Alendronate Sodium, Cholecalciferol	(DC)Fosamax Plus	Postmenopausal osteoporosis; Osteoporosis, in men
10		FOS4FAH7	A11CC05	Alendronate Sodium 70mg, Cholecalciferol 5600IU	Fosamax Plus	Postmenopausal osteoporosis; Osteoporosis, in men
11		FOS4FAH7	M05BB03	Alendronate Sodium 70mg, Cholecalciferol 5600IU	Fosamax Plus	Postmenopausal osteoporosis; Osteoporosis, in men
12		FOS4FAH7	M05BA04	Alendronate Sodium 70mg, Cholecalciferol 5600IU	Fosamax Plus	Postmenopausal osteoporosis; Osteoporosis, in men
13		LAD7LF17	G03CA03	Estradiol	LADIOL 0.60mg	Treats symptoms of menopause and lack of estrogen. Prevents osteoporosis after menopause.
14		MIA3FAA6	H05BA01	Calcitonin, Salmon	Miacalcic 200IU	Treatment of osteoporosis in postmenopausal women
15		OES7LF17	G03CA03	Estradiol	Oestrogel 0.60mg	Treats symptoms of menopause and lack of estrogen. Prevents osteoporosis after menopause.
16		PRO1FAG0	M05BX04	Denosumab	Prolia 60mg	Treats osteoporosis in men and postmenopausal women at high risk for fracture

Figure 5. Image of search result for Example 4 in Clinical Orders Navigator

Example 5: To find the template of Charlson Comorbidity Index

- **Case**: fuzzy search with '%'
- **Order Type**: Diagnosis
- **Mode:** Template
- **Key Words**: CCI%
- **Result**: query result show 1 records

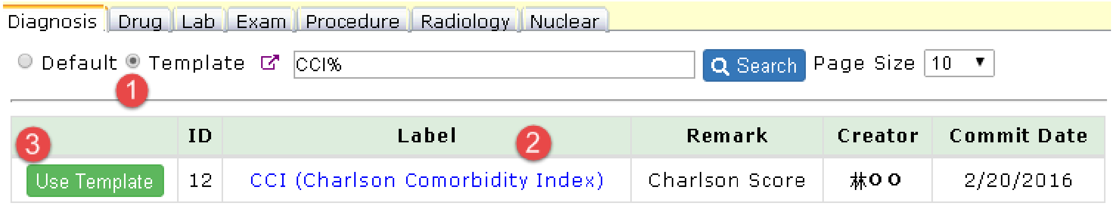

Figure 6. Image of search result for Example 5 in Clinical Orders Navigator

Further Explain:

1. When the researcher selected the Template mode and entered the keyword-"CCI%" and then the Clinical Orders Navigator will search from the Template Library.
2. The search results found a record of the CCI (Charlson Comorbidity Index). The researcher can click the label (blue text) and then it will pop-up a window to show the detail about all orders in the template.
3. The researcher can click the "Use Template" button and it will synchronize all clinical orders to MODULE II: Orders Components from this template.

MODULE II: Orders Components

GOAL: Design a component to record clinical orders which was defined by researchers, and the clinical orders will synchronize to the component from **MODULE I: Clinical Orders Navigator**.

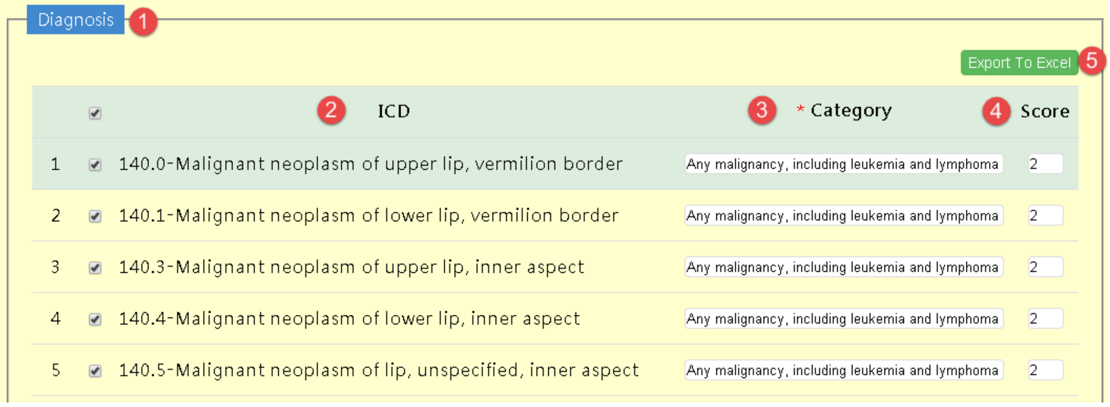

Figure 7. Image of Charlson Comorbidity Index by Orders Components

1. Clinical Order Type:

To record the current clinical order type, such as pharmacy and laboratory.

2. Order Detail:

To record uniquely identifies and description for each clinical order. For example, if the clinical order type is diagnosis, it will record the ICD-9 code and its description.

3. Category:

Module II provide a way to help researchers designing a series of categories, because most of the clinical orders were in a collection.

4. Score:

If you want to give a weight to the clinical orders to calculate some indicators, like

CCI (Charlson Comorbidity Index), and then you can fill in a score based on the literature or guideline. The field is not required.

5. Export to Excel:

Because of **Criteria Components** store clinical domain knowledge, we also implement some batch function, such as export/import form Excel that allow researchers to process batch insert, update and export those criteria, easily.

Case I: [Osteoporotic Fracture] Incident Case

GOAL: Find the patients diagnosed with hip or vertebral fractures and age greater than 50 years old between 2010/01/01 and 2014/12/31. We based on workflow to introduce each stage, as depicted in Figure 10.

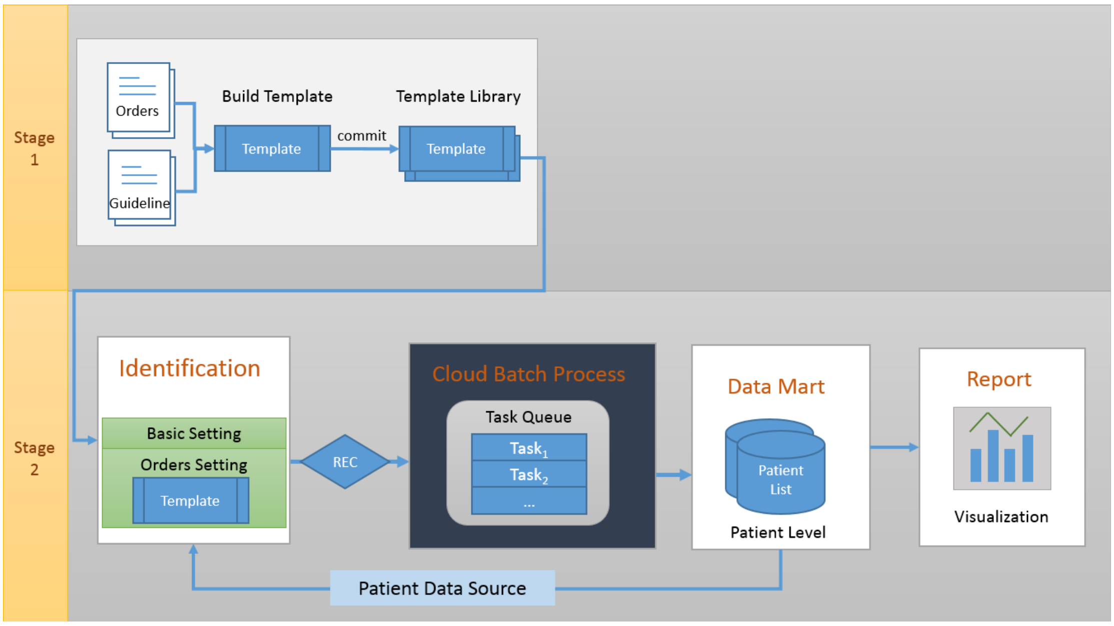

Figure 8. System workflow of NCSS.

Stage 1: Build Template

GOAL: Design a process to create templates for recording clinical orders and categories which based on specific guidelines or research. For example, such as hip or vertebral fractures, including ICD-9-codes 820, 805, and 806 and we cited the guideline to build a template, as depicted in Figure 11.

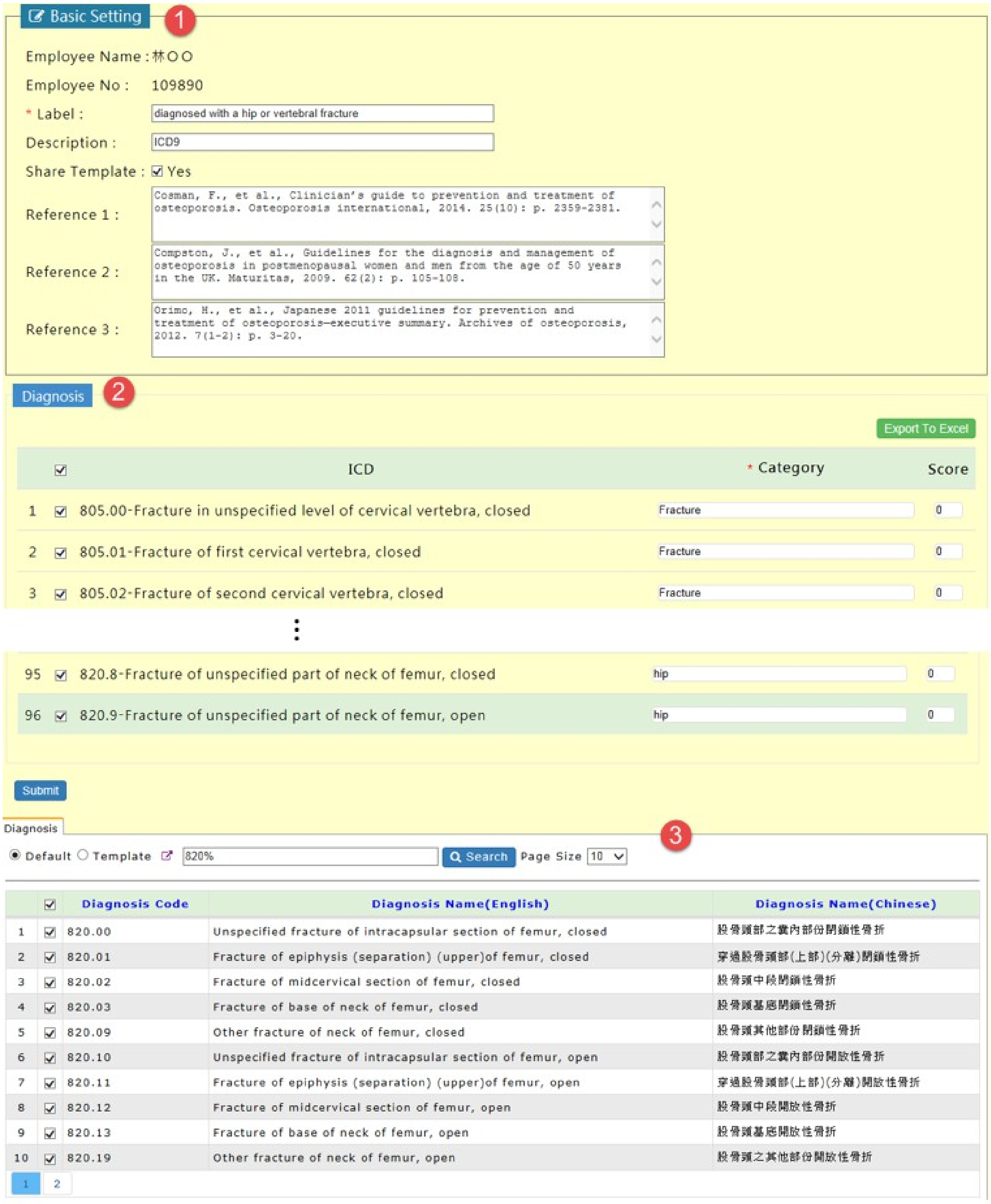

Figure 9. Build a template for recording diagnosis codes and guideline.

1. Basic Setting:

- **Label**: diagnosed with a hip or vertebral fracture
- **Share Template**: Yes
- **Reference 1**: Cosman, F., et al., Clinician's guide to prevention and treatment of osteoporosis. Osteoporosis international, 2014. 25(10): p. 2359-2381.

- **Reference 2**: Compston, J., et al., Guidelines for the diagnosis and management of osteoporosis in postmenopausal women and men from the age of 50 years in the UK. Maturitas, 2009. 62(2): p. 105-108.
- **Reference 3**: Orimo, H., et al., Japanese 2011 guidelines for prevention and treatment of osteoporosis—executive summary. Archives of osteoporosis, 2012. 7(1-2): p. 3-20.

2. Orders Components:

- **Clinical Order Type:** Diagnosis
- **Order Detail:** There are 96 ICD-9-codes including 820, 805, and 806.
- **Category:** ICD-9-codes with 805 and 806 set “Fracture” as a category and ICD-9-codes with 820 set “hip” as a category.
- **Score:** not required. (If you want to give a weight to the clinical orders to calculate some indicators, like CCI (Charlson Comorbidity Index), and then you can fill in a score based on the literature or guideline. The field is not required. More detail please refer to the **MODULE II: Orders Components** on page 10.)

3. Clinical Orders Navigator:

- **Order Type:** Diagnosis
- **Key Words:** ICD-9-codes including 820, 805, and 806.

More detail please refer to the **MODULE I: Clinical Orders Navigator** on page 3.

Stage 2: Patient Identification

GOAL: The stage of Patient identification, which is the matching of the clinical needs to find the patient list. This example demonstrated how to use the template as clinical orders.

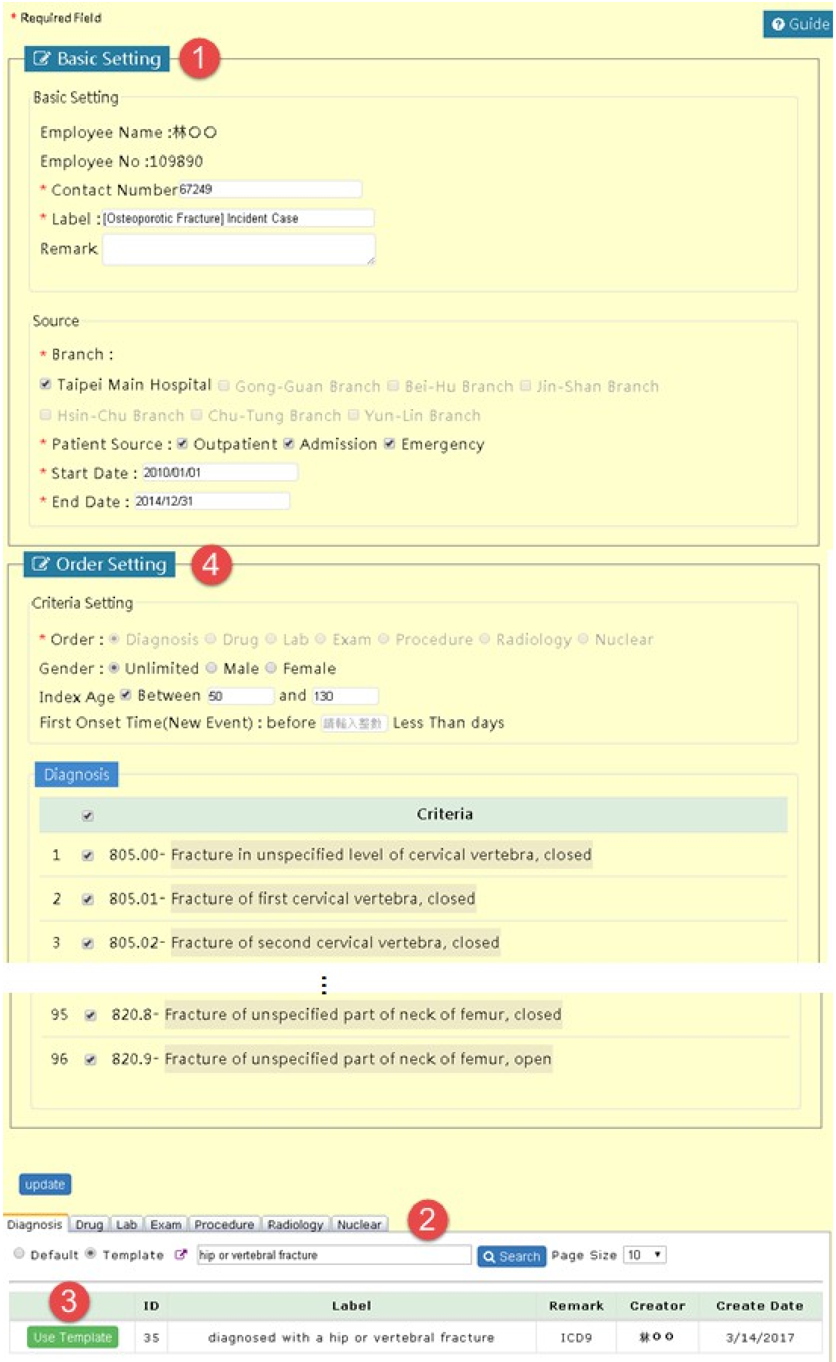

Figure 10. An electronic form for recording the setting of patient identification

Identification

1. Basic Setting:

To define the surveillance topic, and set up the duration of observation from a particular data source, such as outpatient, admission, and emergency.

- **Label:** [Osteoporotic Fracture] Incident Case
- **Branch:** Taipei main hospital
- **Patient source:** outpatient, admission and emergency
- **Start date:** 2009/01/01
- **End date:** 2014/12/31

2. Clinical Orders Navigator:

- **Case**: full text search with ‘Hip or vertebral fracture ’
- **Order Type**: Diagnosis
- **Mode:** Template
- **Key Words**: Hip or vertebral fracture
- **Result**: query result show 1 records

3. Use Template:

If you click the ‘Use Template’ button, this template will synchronize to the orders component. There synchronized 96 diagnosis codes in order setting.

4. Order Setting:

There are two criteria: First, researchers define the 96 diagnosis codes which have been synchronized to the Orders Component. Second, filtering the age between 50 to 130.

REC, Cloud Batch Process and Data Mart

The setting of case “[Osteoporotic Fracture] Incident Case” had been authorized, it was been added a task to Task Queue for cloud batch process in server side. The cloud batch process maintains a Task Queue. Every task will be executed follow First-In-First-Out (FIFO) mechanism in batch server that read the setting of Identification to query eligible patients in IMDB. After completion of the task, the patient list will be stored into database and send a mail notification to researchers. Finally, the case’s status become “Finished” in Data Mart.

ID	Status	Label	Duration	Request Date	Remark	Setting	Report
73	Finished	[Osteoporotic Fracture] Incident Case	1/1/2010 ~ 12/31/2014	7/19/2017			

Figure 21. List the case 73 had been identified in Data Mart.

Report Service

1. View of Characteristics:

The view is demographic summary that help clarify the characteristics for patient list. For example, the descriptive statistics of age, gender, body mass index (BMI), income level, as show as figure 12.

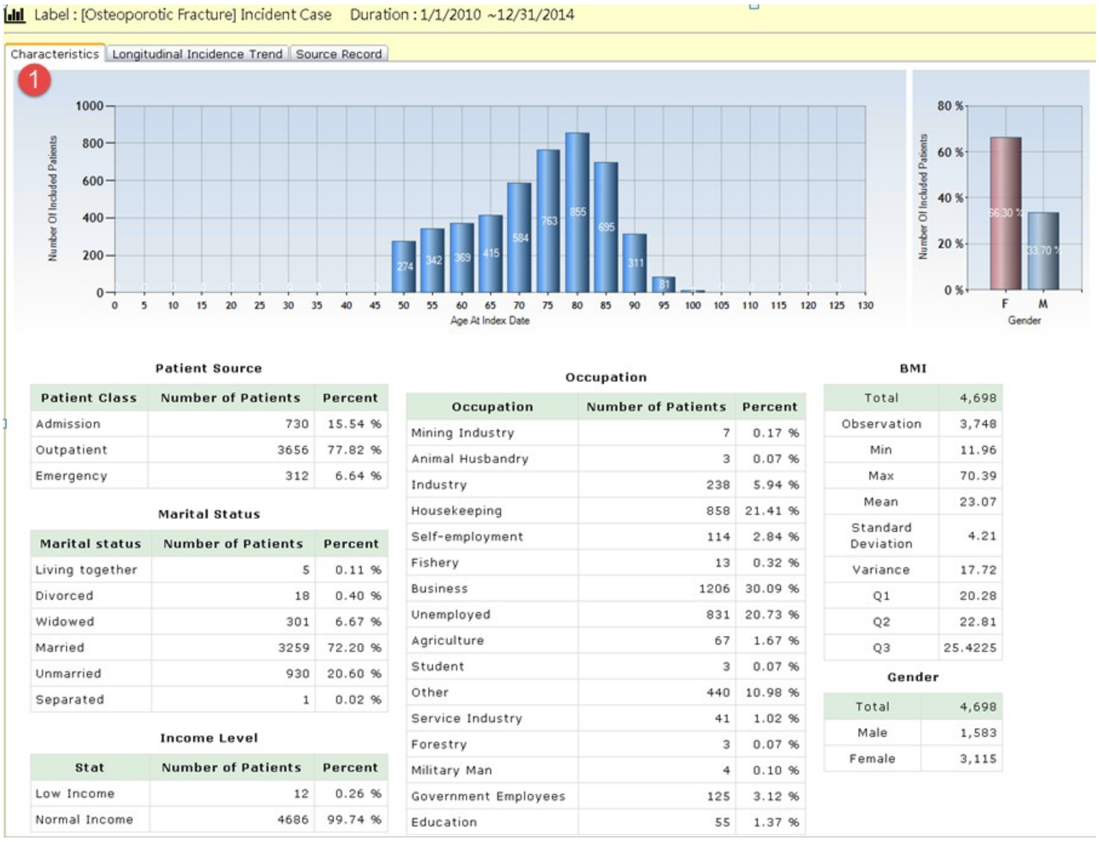

Patient Source

Patient Class	Number of Patients	Percent
Admission	730	15.54 %
Outpatient	3656	77.82 %
Emergency	312	6.64 %

Marital Status

Marital status	Number of Patients	Percent
Living together	5	0.11 %
Divorced	18	0.40 %
Widowed	301	6.67 %
Married	3259	72.20 %
Unmarried	930	20.60 %
Separated	1	0.02 %

Income Level

Stat	Number of Patients	Percent
Low Income	12	0.26 %
Normal Income	4686	99.74 %

Occupation

Occupation	Number of Patients	Percent
Mining Industry	7	0.17 %
Animal Husbandry	3	0.07 %
Industry	238	5.94 %
Housekeeping	858	21.41 %
Self-employment	114	2.84 %
Fishery	13	0.32 %
Business	1206	30.09 %
Unemployed	831	20.73 %
Agriculture	67	1.67 %
Student	3	0.07 %
Other	440	10.98 %
Service Industry	41	1.02 %
Forestry	3	0.07 %
Military Man	4	0.10 %
Government Employees	125	3.12 %
Education	55	1.37 %

BMI

Total	4,698
Observation	3,748
Min	11.96
Max	70.39
Mean	23.07
Standard Deviation	4.21
Variance	17.72
Q1	20.28
Q2	22.81
Q3	25.4225

Gender

Total	4,698
Male	1,583
Female	3,115

Figure 12.view of characteristics in Case I

2. View of Longitudinal Incidence Trend:

The view present incidence trend by time series chart and provide a real-time interactive query by time interval, including monthly, quarterly or yearly, as show in figure 13.

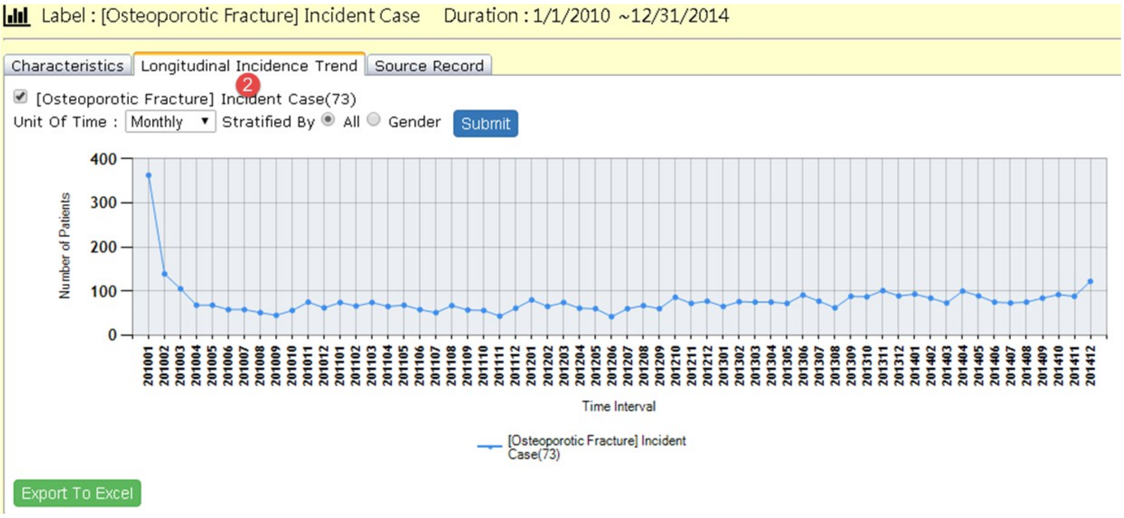

Figure 13. View of Longitudinal Incidence Trend in Case I

3. View of Source Record:

The view present number of include/exclude patients in every Identification process. If the patient list contains hierarchical structure that run the process of identification more than once, the Report Service can track all result of identification in aggregation table, as show as figure 14.

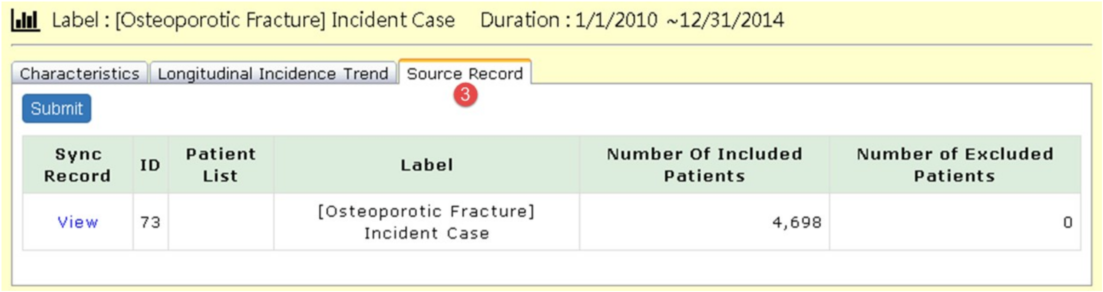

Sync Record	ID	Patient List	Label	Number Of Included Patients	Number of Excluded Patients
View	73		[Osteoporotic Fracture] Incident Case	4,698	0

Figure 14. View of Source Record in Case I

Case II: [Osteoporotic Fracture] excluding malignant neoplasm

GOAL: **To** exclude patients diagnosed with malignant neoplasm within 1 year before index fracture from the patient list based on case 73. This example does not show a similar process; thus, we only demonstrated the Identification process and Report Service. We designed a mechanism for reusing the patient list in the Data Mart. We believe that this identified list of patients should be reused, especially for the design of subgroup study, case control study, or research on a similar context. To better interpret the identification process of each patient list, we designed a hierarchical structure, using which each patient list could be traced back to its patient data source and researchers could compare every patient data source with the characteristics and longitudinal incidence trend in the Report Service. We aimed to describe the advantages of the hierarchical structure, and compare the differences between Cases I and II.

Identification

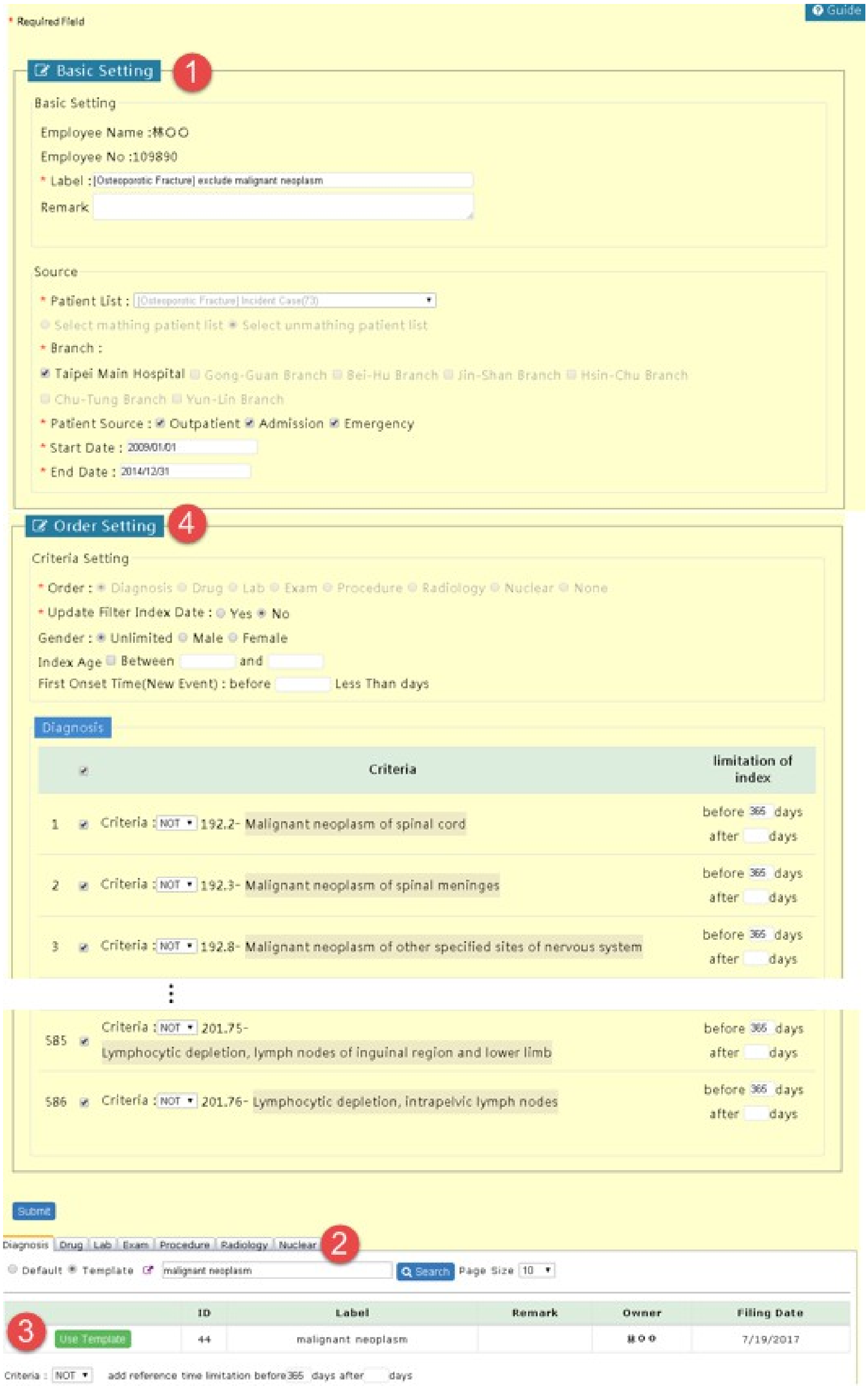

Figure 35. An electronic form for recording the setting of patient identification

1. Basic Setting:

- **Label:** [Osteoporotic Fracture] exclude malignant neoplasm
- **Patient list:** [Osteoporotic Fracture] incident case (case number 73). We designed a mechanism for reusing the patient list in Data Mart, using which researchers could select a patient list from the drop-down list as patient data source for patient identification process.

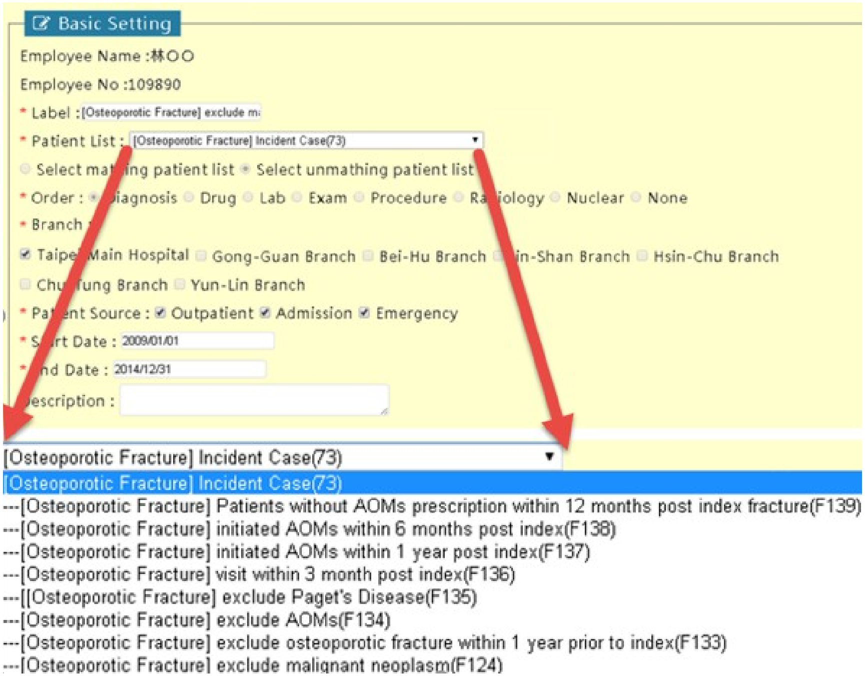

Figure 46. A patient list from the drop-down list as patient data source

- **Order type:** Diagnosis
- **Patient source:** outpatient, admission and emergency
- **Start date:** 2009/01/01
- **End date:** 2014/12/31

2. Clinical Orders Navigator:

Case: full text search with ‘malignant neoplasm’

Order Type: Diagnosis

Mode: Template

Key Words: malignant neoplasm

Result: query result show 1 record

3. Use Template:

If you click the ‘Use Template’ button, this template will synchronize to the orders component. There synchronized 586 diagnosis codes in order setting.

5. Order Setting:

There are two criteria: First, researchers define the 586 diagnosis codes which have been synchronized to the Orders Component and every diagnosis code have 2 status - NOT and before 365 day.

- Include / Exclude : NOT
- Limitation of index : before 365 day

The patient will be excluded when he/she is diagnosed with a malignant neoplasm within one year before the index date (newly diagnosed with a hip or vertebral fracture) by the Cloud Batch Process.

Report Service

1. Characteristics view:

The characteristics view implies the demographic summary of the characteristics that helps clarify them for a patient list. For the case “[Osteoporotic Fracture] excluding malignant neoplasm”, we can realize the descriptive statistics of age, gender, body mass index (BMI), income level, as show in Figure 17. Further, the results of Case I can be compared with those in Figure 12 to understand that the number of males decreased from 1,583 to 1,356, while the number of females decreased from 3,115 to 2,785.

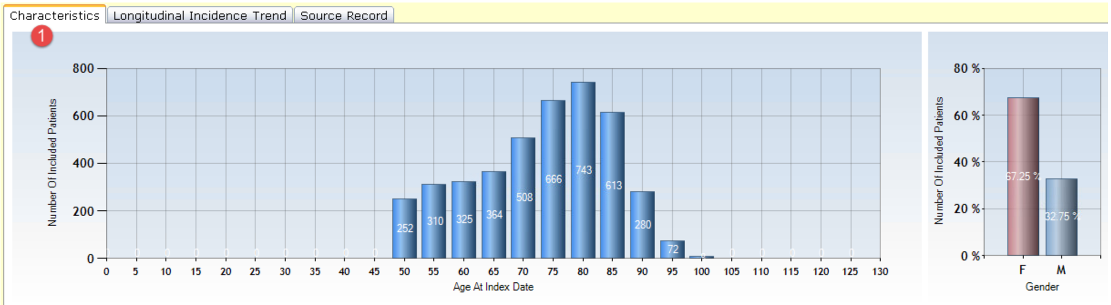

Patient Source

Patient Class	Number of Patients	Percent
Admission	634	15.31 %
Outpatient	3234	78.10 %
Emergency	273	6.59 %

Marital Status

Marital status	Number of Patients	Percent
Living together	4	0.10 %
Divorced	14	0.35 %
Widowed	253	6.38 %
Unmarried	869	21.92 %
Married	2823	71.22 %
Separated	1	0.03 %

Income Level

Stat	Number of Patients	Percent
Low Income	11	0.27 %
Normal Income	4130	99.73 %

Occupation

Occupation	Number of Patients	Percent
Mining Industry	7	0.20 %
Animal Husbandry	3	0.09 %
Industry	208	5.94 %
Housekeeping	764	21.82 %
Self-employment	103	2.94 %
Fishery	11	0.31 %
Business	1046	29.88 %
Unemployed	715	20.42 %
Agriculture	57	1.63 %
Student	3	0.09 %
Other	387	11.05 %
Service Industry	36	1.03 %
Forestry	2	0.06 %
Military Man	3	0.09 %
Government Employees	110	3.14 %
Education	46	1.31 %

BMI

Total	4,141
Observation	3,244
Min	11.96
Max	70.39
Mean	23.17
Standard Deviation	4.26
Variance	18.14
Q1	20.34
Q2	22.86
Q3	25.6150

Gender

Total	4,141
Male	1,356
Female	2,785

Figure 17. View of characteristics case II

2. Longitudinal Incidence Trend view:

Case II has a hierarchical structure, using which each patient list could be traced back to its patient data source. Figure 15 shows two patient lists: Case I "[Osteoporotic Fracture] Incident Case (73)" and Case II "[Osteoporotic Fracture] excluding malignant neoplasm". The end user could compare every patient list by using the longitudinal incidence trend at specific intervals such as monthly, quarterly, and yearly. We compared Cases I and II quarterly with respect to the Longitudinal Incidence Trend view and stratified them based on gender, as shown in Figure 18.

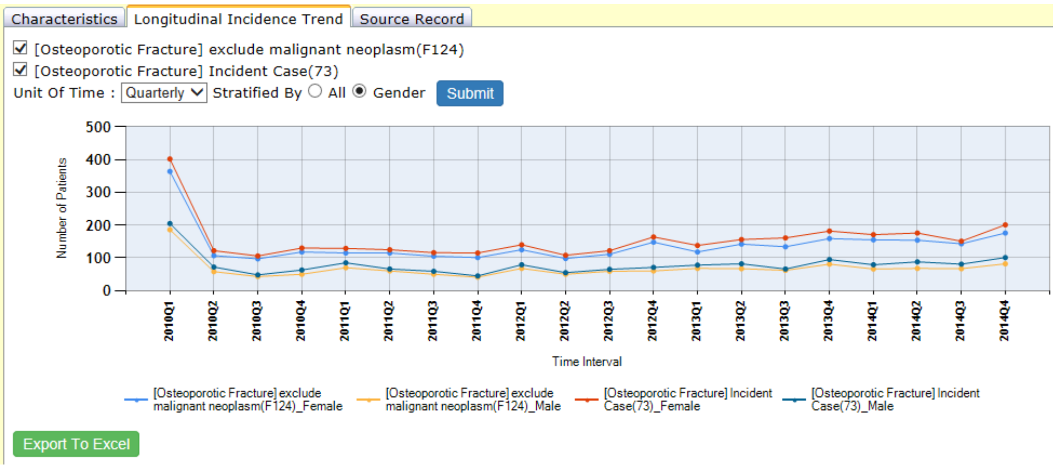

Figure 18. Compare with Case I and Case II in View of Longitudinal Incidence Trend by Quarterly and stratified by gender.

3. Source Record view:

Here, we describe the advantages of the hierarchical structure in the Source Record view. As Case II contains a hierarchical structure that runs the process of identification more than once, the Report Service can track all the results of identification in the aggregation table. Finally, we can obtain a source record report. The Source Record view presents the number of patients included/excluded in every Identification process, as shown in Figure 19.

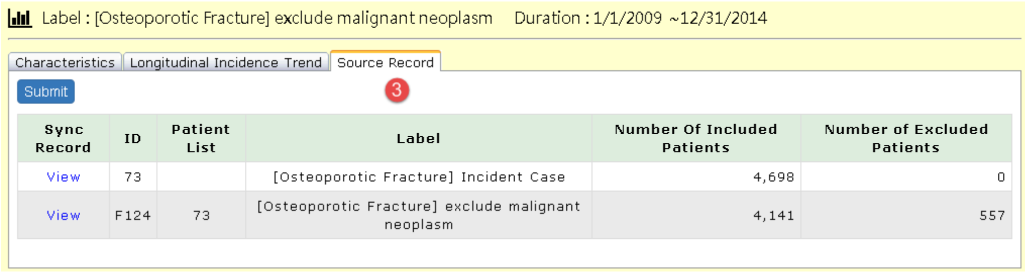

Sync Record	ID	Patient List	Label	Number Of Included Patients	Number of Excluded Patients
View	73		[Osteoporotic Fracture] Incident Case	4,698	0
View	F124	73	[Osteoporotic Fracture] exclude malignant neoplasm	4,141	557

Figure 19. View of Source Record in case II

Finally, a cohort study flow was developed based on the results (Figure 20).

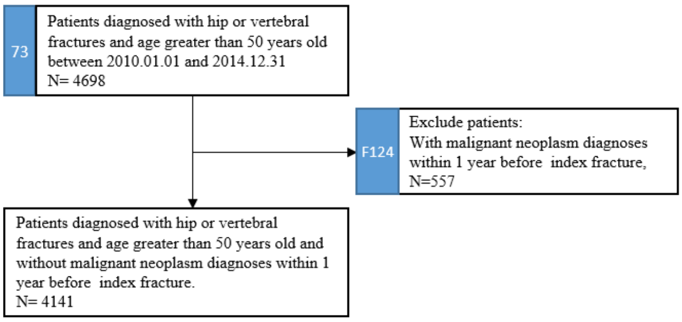

Figure 20. A cohort study flow based on the results of Case II
